# The Association Between Sedentary Behavior and Cardiovascular Disease Risk: An Analysis Based on NHANES Data

**DOI:** 10.1155/cdr/6940329

**Published:** 2026-04-06

**Authors:** Heming Huang, Zhe Zhao, Zemou Yu, Fan Feng, Zaifeng Zhang, Bingge Fan, Ling-bing Meng

**Affiliations:** ^1^ Geriatric Department, Shenzhen People’s Hospital, The Second Clinical Medical College of Jinan University, The First Affiliated Hospital of Southern University of Science and Technology, Shenzhen, Guangdong, China, jnu.edu.cn; ^2^ Department of Pharmacy, Peking University Third Hospital, Beijing, China, puh3.net.cn; ^3^ Institute for Drug Evaluation, Peking University Health Science Center, Beijing, China, bjmu.edu.cn; ^4^ Department of Neurology, Beijing Children’s Hospital, Capital Medical University, National Center for Children’s Health, Beijing, China, bch.com.cn; ^5^ Department of Neurology, Beijing Hospital, National Center of Gerontology, Institute of Geriatric Medicine, Chinese Academy of Medical Sciences, Beijing, China, cacms.ac.cn; ^6^ Center of Laboratory Medicine, State Key Laboratory of Cardiovascular Disease, Beijing Key Laboratory for Molecular Diagnostics of Cardiovascular Diseases, Fuwai Hospital, Chinese Academy of Medical Sciences & Peking Union Medical College/National Center for Cardiovascular Diseases, Beijing, China, fuwaihospital.org; ^7^ Department of Endocrinology, The Fourth Hospital of Hebei Medical University, Shijiazhuang, China, hebmu.edu.cn; ^8^ Cardiometabolic Medicine Center, National Clinical Research Center for Cardiovascular Diseases, Fuwai Hospital, National Center for Cardiovascular Diseases, Chinese Academy of Medical Sciences and Peking Union Medical College, Beijing, China, cacms.ac.cn; ^9^ Department of Cardiology, National Clinical Research Center for Cardiovascular Diseases, Fuwai Hospital, National Center for Cardiovascular Diseases, Chinese Academy of Medical Sciences and Peking Union Medical College, Beijing, China, cacms.ac.cn; ^10^ State Key Laboratory of Cardiovascular Disease, Beijing, China; ^11^ Translational Medical Center, Weifang Second People’s Hospital, Weifang, China, wfdeyy.com

**Keywords:** cardiovascular disease, CVD, NHANES, risk factors, sedentary behavior

## Abstract

**Background:**

Sedentary behavior has become a major global public health challenge and is closely associated with cardiovascular disease (CVD). Sedentary behavior not only increases the risk of various chronic diseases but also poses significant threats to cardiovascular health. Existing studies suggest that sedentary behavior is an independent risk factor for CVD, but the underlying mechanisms across different populations remain inadequately explored.

**Methods:**

This study analyzed data from 31,034 participants in the National Health and Nutrition Examination Survey (NHANES) to investigate the relationship between sedentary behavior and CVD. Standardized questionnaires were used to collect sociodemographic and health information. Statistical analyses, including weighted t‐tests, forest plots, logistic regression, and restricted cubic spline regression, were employed to examine the effects of sedentary time on CVD and related influencing factors.

**Results:**

The findings indicated that prolonged sedentary time was significantly associated with an increased risk of CVD, particularly among individuals aged 60 and older (OR = 17.297, *p* < 0.001) Factors such as age, gender, and hyperlipidemia played a critical role in the relationship between sedentary behavior and CVD. Restricted cubic spline regression analysis revealed that CVD risk increased markedly after 250 min of sedentary time, with a pronounced rise after 750 min. Multivariate regression analysis also confirmed the negative impact of sedentary behavior on cardiovascular health.

**Conclusion:**

Sedentary behavior is a significant independent risk factor for CVD, and reducing sedentary time is associated with a lower CVD risk. Enhancing public awareness of the hazards of sedentary behavior and implementing effective interventions, especially for the elderly, may contribute to improved cardiovascular health.

## 1. Introduction

In recent years, sedentary behavior has emerged as a significant global public health challenge. Sedentary behavior is generally defined as activities with very low energy expenditure (less than 1.5 METs), such as prolonged sitting or lying down while awake [1, 2]. With advancements in technology and urbanization, changes in work and lifestyle patterns have led to increasingly sedentary habits. In the United States, for instance, adults now spend an average of 6–8 h per day in sedentary activities [3, 4].

Studies have shown that sedentary behavior is closely associated with various health issues, including cardiovascular disease (CVD), diabetes, and cancer, as well as their related mortality rates. There is a significant positive correlation between sedentary time and all‐cause mortality (ACM), cardiovascular mortality, and cancer mortality, highlighting sedentary behavior as an independent risk factor [5, 6].

Specifically, sedentary behavior and physical inactivity are among the primary modifiable risk factors for global CVD and ACM. Physical activity and exercise training (ET) are crucial for improving cardiorespiratory health, particularly in preventing chronic noncommunicable diseases, including CVD [1]. Further research emphasizes that increasing physical activity and reducing sedentary time not only enhance cardiovascular health but also significantly reduce the risk of CVD [7].

CVD remains one of the leading causes of death worldwide, with high morbidity and mortality rates [8]. According to the American Heart Association, CVD accounts for approximately 17.3 million deaths annually [9, 10]. Additionally, CVD poses a substantial economic burden. A study conducted in the European Union reported that CVD incurs an annual cost of €282 billion, of which 55% is attributed to healthcare and long‐term care expenses, 17% to productivity losses, and 28% to informal care costs [11].

Sedentary behavior is closely linked to multiple risk factors for cardiometabolic diseases, including elevated blood pressure, vascular dysfunction, lipid metabolism abnormalities, insulin resistance, endothelial dysfunction, and low‐grade inflammation [12, 13]. Sedentary behavior may exacerbate atherosclerosis and the onset of CVD by slowing blood circulation, inducing metabolic abnormalities (such as decreased insulin sensitivity and lipid dysregulation), and triggering chronic low‐grade inflammation [5, 14, 15]. Studies have demonstrated that increasing physical activity, particularly by reducing sedentary behavior, can significantly lower blood pressure and, consequently, reduce the risk of CVD [16].

Moreover, studies have shown that women who engage in prolonged sedentary behavior, especially with extended sedentary bouts, have a higher risk of CVD. This finding suggests that reducing total sedentary time and avoiding prolonged sitting periods can help lower the risk of CVD [17, 18]. Randomized clinical trials indicate that reducing sedentary behavior can improve certain cardiovascular health indicators. Although no significant effects were observed in reducing blood pressure, decreasing sedentary time during work showed a beneficial impact on diastolic blood pressure (DBP) [19]. These findings further support the strong association between sedentary behavior and cardiovascular health, highlighting that reducing sedentary behavior could be an important public health intervention for improving cardiovascular health.

Despite extensive research on the relationship between sedentary behavior and CVD, the specific mechanisms of its impact on different populations, including age, gender, and socioeconomic status (SES), remain underexplored. Existing studies have predominantly focused on developed countries, leaving the effects of sedentary behavior under different cultural and lifestyle contexts insufficiently examined. More importantly, the health impact of sedentary behavior may be moderated by individual characteristics (e.g., age and gender) and environmental factors (e.g., occupational type and SES) [20]. Research has found that low levels of physical activity and high sedentary behavior in low SES groups are more strongly associated with CVD and ACM, suggesting that SES may play a critical moderating role in the relationship between sedentary behavior and health risks [21].

To address gaps in existing research and further verify the specific impact of sedentary behavior on CVD, this study integrates a wide range of sociodemographic and health‐related data based on the National Health and Nutrition Examination Survey (NHANES). The study is aimed at analyzing the relationship between sedentary behavior and CVD comprehensively, exploring health disparities across different populations, and confirming the role of sedentary behavior as an independent risk factor for CVD. The findings of this analysis will provide a scientific basis for developing public health policies and offer valuable insights for designing interventions to reduce sedentary behavior.

## 2. Materials and Methods

### 2.1. Clinical Data

The data of this study come from the NHANES. NHANES is a national cross‐sectional survey conducted jointly by the National Center for Health Statistics (NCHS) and the Centers for Disease Control and Prevention (CDC). The survey is aimed at collecting nationally representative data of the US civilian population, covering multiple aspects such as health status, lifestyle, and nutritional status. During the data collection process, all participants were approved by the NCHS Ethics Review Committee and signed an informed consent form before interviews and examinations. The study population includes all respondents participating in NHANES, covering Americans of different ages, genders, races, and ethnic backgrounds. By analyzing these data, we can have a more comprehensive understanding of the health status and disease burden of the US population, providing a scientific basis for the formulation of public health policies and intervention measures.

To ensure the quality and reliability of the data, the NHANES survey adopted a strict data collection and processing procedure. The survey content includes face‐to‐face interviews, physical examinations, and laboratory tests and is carried out by professional survey personnel. After data cleaning and verification, it is released in the form of public data files for researchers to use.

The NHANES data used in this study are publicly accessible deidentified data. All original NHANES surveys were approved by the Research Ethics Review Board of the NCHS in the United States, and all participants provided written informed consent prior to the survey. This study is a secondary analysis of existing publicly available data and has been approved by the Ethics Committee of Fuwai Hospital, Chinese Academy of Medical Sciences, Approval Number 2025‐2847.

This study will use NHANES data to deeply analyze the relevant risk factors of CVDs and explore the health status differences among different populations. The results of the study will provide important reference for the prevention and control of CVDs and help improve the health level of the US population.

### 2.2. Data Collection

This study is aimed at exploring the impact of sedentary behavior on CVDs, while considering a series of potential covariates. These variables, based on previous literature and substantive reasoning, are considered to have potential associations with sedentary behavior and CVD rates. Standardized questionnaires were used to collect information on various factors, including age, gender, race, education level, marital status, poverty–income ratio (PIR), body mass index (BMI), waist circumference, smoking status, alcohol use, and medical comorbidities (such as hypertension, hyperlipidemia, and diabetes). For missing values in the data, we used interpolation through R software for supplementation.

The data for this study were sourced from the US NHANES, including a total of 31,034 participants. Based on the analysis requirements, we strictly screened the study sample in the following order: excluded participants under 20 years old (*n* = 5,247): NHANES participants must be 20 years or older to ensure the study population consists of adults; excluded participants with missing sedentary behavior data (*n* = 3812); excluded participants with missing CVD status data (*n* = 6119); excluded participants with missing key covariate data, including demographic characteristics (sex, race, and education level), lifestyle factors (smoking, alcohol consumption, and physical activity), health indicators (BMI, blood pressure, blood lipids, and history of diabetes), and SES (family income ratio) (*n* = 9304). Ultimately, 6552 participants who met all inclusion criteria and had complete key variable data were included in the final cross‐sectional analysis.

The diagnosis of CVDs was based on the self‐reported questionnaire results of participants, including affirmative answers to the NHANES multiple‐choice questions (MCQs) about “whether they have ever been told that they have (congestive) heart failure, coronary heart disease, angina, myocardial infarction (MI), or stroke” or whether they use cardiovascular drugs.

Hypertension was defined as participants self‐reporting a diagnosis of hypertension, an average systolic blood pressure (SBP) ≥ 130 mmHg or an average DBP ≥ 80 mmHg, or taking antihypertensive drugs. Hyperlipidemia was defined according to the standards of the National Cholesterol Education Program (NCEP) Adult Treatment Panel III (ATP III), including total cholesterol ≥ 200 mg/dL, triglycerides ≥ 150 mg/dL, high‐density lipoprotein (male < 40 mg/dL, female < 50 mg/dL) or low‐density lipoprotein ≥ 130 mg/dL, and individuals reporting the use of cholesterol‐lowering drugs. Based on existing public health guidelines and the exploratory purpose of this study, we categorized sedentary time into two types for analysis: (1) normal sedentary: ≤ 4 h/day and long‐term sedentary: > 4–≤ 8 h/day and (2) excessive sedentary: > 8 h/day. All sedentary time data were self‐reported by the participants.

By comprehensively analyzing these data, this study is aimed at revealing the association between sedentary behavior and CVDs and exploring the role of other related factors in this relationship.

### 2.3. Statistical Analysis

All statistical analyses were conducted in accordance with the guidelines of the CDC and utilized sampling weights to generate nationally representative prevalence estimates for the noninstitutionalized US population. Continuous variables are presented as mean ± standard deviation (SD), while categorical variables are reported as numbers and percentages. For normally distributed measurement data, the mean ± SD(*x* ± *s*) is used for description, and the *t*‐test is employed to assess differences between groups. For data not normally distributed, the median and interquartile range (M[P25, P75]) are used for description. Weighted *t*‐tests (for continuous variables) or weighted chi‐square tests (for categorical variables) are used to compare differences between two groups. Forest plots are utilized to analyze the association between sedentary time and CVD. Logistic regression and interaction tests are used to confirm the stability of the association between sedentary time and CVD across different groups; restricted cubic spline (RCS) regression is used to explore the nonlinear relationship between sedentary time and CVD.

## 3. Results

### 3.1. Baseline Characteristics of Participants

This study collected survey data from 31,034 participants. After excluding those lacking outcome or exposure data and further excluding participants who did not provide complete information on BMI, education level, marital status, smoking, and alcohol consumption, a total of 6552 participants were included in the final analysis. The distribution of participant characteristics grouped by the presence or absence of CVD showed significant differences in several clinical variables between participants with CVD (*n* = 222) and those without (*n* = 6330) in the preliminary analysis. Among those who were sedentary, the number of individuals with CVD was 212 (3.20%), compared to 10 (0.20%) in the nonsedentary group, indicating a correlation between prolonged sedentary time and an increased risk of CVD (*p* < 0.001). The age group of 60 and above in the CVD group had the highest significance with 152 (2.30%) (*p* < 0.000). In terms of gender distribution, the proportion of males in the CVD group was 2.60%, significantly higher than the proportion of females in the same group at 0.80% (*p* < 0.000). Regarding marital status, the proportion of unmarried individuals in the CVD group was 2.10%, significantly higher than the proportion of married/cohabitating individuals in the same group at 0.20% (*p* < 0.000). In terms of education level, the proportion of individuals with a college degree or above in the CVD group was 0.90%, significantly higher than the 0.50% in the other two groups with CVD (*p* = 0.002). Regarding income, the proportion of low‐income individuals in the CVD group was 1.80%, significantly higher than the proportions of medium and high‐income individuals in the same group at 0.80%. In terms of chronic diseases, the incidence of pulse rate in the CVD group was 3.10%, significantly higher than the 0.30% in the group with CVD (*p* < 0.000). In terms of nutritional intake, the calcium level in the CVD group (56.48 ± 88.01 mg) was significantly lower than in the group without CVD (76.92 ± 142.343 mg) (*p* = 0.034), and the protein level (3.12 ± 4.98 mg) was also lower than in the group without CVD (4.74 ± 9.03 mg)(*p* = 0.080). Moreover, the hemoglobin value in the CVD group (90.17, IQR: 77.79~109.39) was significantly higher than in the group without CVD (74.26, IQR: 63.65~85.75) (*p* < 0.000). Regarding lifestyle habits, the proportion of never drinkers in the CVD group was 0.30%, significantly lower than the 12.10% in the group without CVD (*p* = 0.492), but the proportion of never smokers in the former (1.10%) was significantly lower than in the latter (53.20%) (*p* < 0.000). Details are presented in Table 1.

**Table 1:** Baseline characteristics.

Variable		Cardiovascular		*p* value
		Yes (*n* = 222)	No (*n* = 6330)	
Age	20–40 years old	12 (0.20%)	2705 (41.30%)	< 0.001
	40–60 years old	58 (0.90%)	2099 (32.00%)	
	> 60 years old	152 (2.30%)	1526 (23.30%)	
Gender	Male	168 (2.60%)	3042 (46.40%)	< 0.001
	Female	54 (0.80%)	3288 (50.20%)	
Education level	Less than 9th grade	34 (0.50%)	574 (8.80%)	0.002
	9th–11th grade	33 (0.50%)	974 (14.90%)	
	High school	62 (0.90%)	1482 (22.60%)	
	College or above	93 (1.40%)	3300 (50.40%)	
Marial status	Never married	135 (2.10%)	3919 (59.80%)	< 0.001
	Married/living with partner	11 (0.20%)	1175 (17.90%)	
	Widowed/divorced/separated	76 (1.20%)	1236 (18.90%)	
Income status	Low income	121 (1.80%)	2831 (43.20%)	0.015
	Middle income	51 (0.80%)	1716 (26.20%)	
	High income	50 (0.80%)	1783 (27.20%)	
BMI	< 20.5	8 (0.10%)	384 (5.90%)	0.296
	20.5–40.0	199 (3.00%)	5564 (84.90%)	
	> 40.0	15 (0.20%)	10 (5.80%)	
Sedentary	Normal sitting duration or prolonged sitting	10 (0.20%)	523 (8.00%)	< 0.001
	Excessively long sitting duration	212 (3.20%)	2807 (88.60%)	
ALT (U/L)		23.94 ± 12.220	24.64 ± 20.475	0.615
Creatinine		90.17 (77.79~109.39)	74.26 (63.65~85.75)	< 0.001
Hemoglobin		14.34 ± 1.54	14.19 ± 1.49	0.145
Energy intake		1636 (1265~2144)	1966.50 (1466~2622)	< 0.001
Calcium		56.48 ± 88.01	76.92 ± 142.34	0.034
Protein		3.12 ± 4.98	4.74 ± 9.03	0.080
Fiber		1.06 ± 2.69	1.06 ± 2.40	0.968
Magnesium		23.70 ± 31.98	25.70 ± 32.89	0.373
Sodium		163.46 ± 290.18	201.89 ± 356.38	0.112
Potassium		177.89 ± 199.08	201.30 ± 269.22	0.199
Total fat		2.91 ± 6.90	4.10 ± 8.95	0.490
Saturated fat		0.84 ± 2.32	1.31 ± 3.11	0.028
Cholesterol		18.21 ± 88.87	29.90 ± 102.20	0.093
Diabetes	Yes	57 (0.90%)	577 (8.80%)	< 0.001
	No	165 (2.50%)	5753 (87.80%)	
Hypertension	Yes	2 (0.00%)	20 (0.30%)	0.170
	No	220 (3.40%)	6310 (96.30%)	
Hyperlipidemia	Yes	48 (0.70%)	2557 (39.00%)	< 0.001
	No	174 (2.70%)	3773 (57.60%)	
Smoke	Never	70 (1.10%)	3483 (53.20%)	< 0.001
	Former	102 (1.60%)	1471 (22.50%)	
	Current	50 (0.80%)	1376 (21.00%)	
Drink	Never drank	22 (0.30%)	793 (12.10%)	0.492
	Occasionally drank	36 (0.50%)	847 (12.90%)	
	Regularly drank	164 (2.50%)	4663 (71.20%)	
	Heavy drinker	0 (0.00%)	16 (0.20%)	
Pulse	Yes	200 (3.10%)	6194 (94.50%)	< 0.001
	No	22 (0.30%)	136 (2.10%)	
Sleep quality	Normal sleep duration	114 (1.70%)	3795 (57.90%)	0.003
	Insufficient sleep	97 (1.50%)	2394 (36.50%)	
	Excessive sleep	11 (0.20%)	141 (2.20%)	

Abbreviations: ALT, alanine aminotransferase; BMI, body mass index; U/L, units per liter.

### 3.2. Forest Plot Analysis of the Association Between Sedentary Behavior and CVD

**Figure 1:**
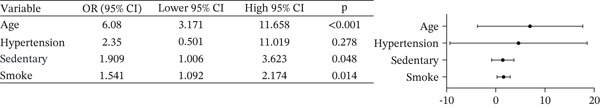
Forest plot of risk factors for cardiovascular disease. This forest plot presents the odds ratios (ORs) and 95% confidence intervals (CIs) for key risk factors associated with cardiovascular disease. Age is identified as the strongest risk factor, with an OR of 6.08 (95% CI: 3.171–11.658, *p* < 0.001). Smoking also shows a significant association, with an OR of 1.541 (95% CI: 1.092–2.174, *p* = 0.014). Sedentary behavior is a statistically significant contributor, with an OR of 1.909 (95% CI: 1.006–3.623, *p* = 0.048). Hypertension demonstrates a nonsignificant trend, with an OR of 2.35 (95% CI: 0.501–11.019, *p* = 0.278).

A forest plot was utilized to display the association between four variables (age, hypertension, sedentary behavior, and smoking) and the risk of CVD. Each variable is accompanied by a corresponding odds ratio (OR) and its 95% confidence interval (CI), along with the respective *p* value. This analysis revealed associations between age, sedentary behavior, smoking, and the risk of CVD. Age showed a significant positive correlation with the risk of CVD (OR = 6.08, *p* < 0.001), indicating that older age was associated with a higher risk of disease. Sedentary behavior exhibited a certain degree of positive correlation with the risk of CVD (OR = 1.909, *p* = 0.048), indicating that the odds of having CVD in the group with the longest sitting time were 1.909 times that of the reference group. Smoking demonstrated a positive trend with CVD, with a *p* value of 0.014, implying that smokers had a higher odds of disease. Particularly, sedentary behavior, despite its relatively small OR value, its statistical significance alerts us to the importance of not overlooking its association with CVD risk assessment. Details are presented in Figure 1.

### 3.3. RCS Regression Analysis of the Nonlinear Relationship Between Sedentary Time and CVD

**Figure 2:**
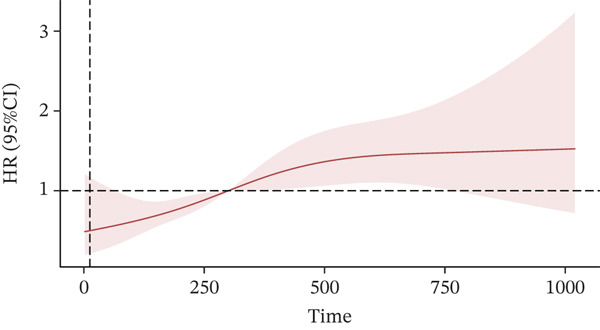
Association between sedentary behavior and cardiovascular disease risk. This graph depicts the relationship between sedentary time and the hazard ratio (HR) for cardiovascular disease, with 95% confidence intervals. The *x*‐axis represents sedentary time (hours per day), and the *y*‐axis shows the HR and its 95% CI. As sedentary time increases, the HR also rises, indicating a positive association. The red line represents the average HR, while the shaded area illustrates the 95% CI, reflecting the uncertainty of the estimate.

In the RCS regression analysis, after adjusting for potential confounding variables, the figure displays the hazard ratio (HR) values and their 95% CIs at different time points. The chart shows a distinct fluctuation trend of HR values over time. The study indicates a significant association between prolonged sedentary time and an increased risk of CVD. The HR showed a gradual increase with longer sedentary time, suggesting an association between longer sedentary duration and higher CVD risk. Specifically, when sedentary time exceeds 250 min, the HR begins to significantly rise above the baseline level of 1.0, indicating an increased risk of cardiovascular events. Between 750 and 1000 min, the HR even surpasses 2.0 and approaches 4.0, highlighting a strong association between long‐term sedentary behavior and adverse cardiovascular outcomes. Moreover, although the 95% CI fluctuates, it does not include the HR level of 1.0 in most time periods, further confirming the reliability of the aforementioned observed association. Therefore, these results are consistent with the hypothesis that reducing sedentary time and increasing physical activity may be important for the prevention of CVD. Details are presented in Figure 2.

### 3.4. Logistic Regression and Interaction Testing for the Stability of the Association Between Sedentary Time and Other Factors With CVD

To carefully examine the reliability and robustness of the relationship between sedentary time and CVD, logistic regression was conducted. The summarized results of the logistic regression are presented in Table 2. The main findings indicate that age, gender, and hyperlipidemia are significantly associated with CVD. An interaction was observed among age groups, suggesting that the association of sedentary behavior on the risk of CVD varied across different age groups, with the odds of CVD being 5.959 times higher in the 40–60 age group compared to the 20–40 age group. Other variables (marital status, education level, BMI, diabetes, hypertension, degree of depression, and sleep duration) were not significantly associated with CVD. Interaction analysis results: The *p* value for the interaction between age and sedentary behavior was 0.489, indicating that the effect of age on the association between sedentary behavior and CVD is not significant. The *p* value for the interaction between hyperlipidemia and sedentary behavior was 0.056, indicating that the effect of hyperlipidemia on the association between sedentary behavior and CVD is not significant. Sedentary behavior is an independent risk factor for CVD, and its impact is more pronounced in the elderly population. It is recommended to take measures to reduce sedentary behavior to lower the risk of CVD. Details are presented in Table 2.

**Table 2:** Validation of the association between sedentary behavior and cardiovascular disease through logistic regression analysis.

Variable		OR (95% CI)	*p* value	*p* for interaction
Age	40–60 years old	5.959 (3.108–11.423)	< 0.001	0.489
	> 60 years old	17.297 (9.086–32.931)	< 0.001	
Gender	Male	0.310 (0.218–0.440)	< 0.001	0.128
Marial status	Married/living with partner	0.728 (0.337–1.406)	0.344	0.919
	Widowed/divorced/separated	1.363 (0.989–1.878)	0.058	
Education	9th–11th grade	0.855 (0.508–1.442)	0.557	0.279
	High school	1.166 (0.732–1.856)	0.519	
	College or above	0.982 (0.620–1.554)	0.938	
BMI		1.028 (1.005–1.051)	0.016	0.855
Hyperlipidemia	No	0.447 (0.317–0.629)	< 0.001	0.056
Diabetes	No	1.244 (0.877–1.766)	0.221	0.669
Hypertension	No	2.202 (0.471–10.295)	0.316	0.999
Depression	Moderate depression	2.285 (0.457–11.426)	0.314	0.895
	Severe depression	0.947 (0.273–3.286)	0.932	
Sleep	Insufficient sleep	1.275 (0.953–1.707)	0.102	0.680
	Excessive sleep	1.971 (0.977–3.977)	0.058	

### 3.5. Multivariate Linear Regression Analysis of the Impact of Sedentary Time on CVD

The multivariate linear regression model indicated that marital status, BMI, sedentary time, and educational level significantly affect cardiovascular health. Marital status (*B* = 0.008, *p* = 0.005), BMI (*B* = 0.001, *p* = 0.003), and prolonged sedentary time (*B* = 0.019, *p* = 0.023) were all associated with an increased cardiovascular risk. In contrast, higher educational level (*B* = −0.008, *p* = 0.001) was associated with a decreased risk. The effects of alcohol consumption and hypertension were not significant. VIF values were all close to 1, indicating minimal multicollinearity issues. In summary, improving lifestyle habits, controlling body weight, and enhancing educational level are crucial for cardiovascular health. Details are presented in Table 3.

**Table 3:** Multiple linear regression model for cardiovascular disease.

	*B*	SE	*t*	*p* value	VIF
Marial status	0.008	0.003	2.783	0.005	1.002
BMI	0.001	0.000	2.977	0.003	1.009
Drinking	0.003	0.003	1.044	0.296	1.014
Hypertension	0.048	0.039	1.239	0.215	1.003
Sedentary	0.019	0.008	2.270	0.023	1.021
Education	−0.008	0.002	−3.368	0.001	1.030

## 4. Discussion

In this cross‐sectional study, we found that sedentary behavior was significantly associated with a higher risk of CVD. Regarding the novelty of our findings, this study provides a comprehensive analysis of the association between sedentary behavior and CVD risk across different age groups, educational levels, and in the context of other cardiovascular risk factors, offering a more in‐depth and multifaceted understanding of this relationship. Moreover, it emphasized the importance of considering the interaction between sedentary behavior and other factors when formulating public health strategies, which is a relatively novel perspective compared to previous studies. From the perspective of public health significance, reducing sedentary behavior has far‐reaching implications. Our results suggest that interventions aimed at reducing sedentary time might not only be associated with a lower incidence of CVD but could also contribute to improving the overall cardiovascular health of the population, thereby potentially reducing the burden on the healthcare system and enhancing the quality of life for individuals. Regarding the use of artificial intelligence tools, no artificial intelligence tools were used in the preparation of this manuscript, from data collection and analysis to the writing and revision processes.

Individuals with prolonged sedentary time showed a markedly higher prevalence of CVD compared to those with shorter sedentary time (3.2% vs. 0.2%, *p* < 0.001). These findings align with previous studies, supporting the role of sedentary behavior as an independent risk factor for CVD.

Research has shown that individuals engaged in sedentary work have a 34% higher risk of CVD‐related mortality (HR 1.34, 95% CI 1.22–1.46) [22], further validating the significant association of sedentary behavior on CVD risk. This suggests that sedentary behavior is not only adversely associated with cardiovascular health but also increases the risk of mortality. Additionally, studies utilizing accelerometer‐based measurements of sedentary behavior found that individuals with sedentary time exceeding 10.6 h per day face significantly elevated risks of heart failure and CVD mortality. Reducing sedentary time has been shown to significantly lower health risks. Increasing leisure‐time physical activity, in particular, can effectively mitigate the health risks associated with prolonged sedentary behavior, especially for those with chronic sedentary habits [23].

This study also reveals that the impact of sedentary behavior on CVD risk varies significantly across different populations. Specifically, the older the individual, the more pronounced the effect of sedentary behavior on CVD risk (OR = 5.959 for individuals aged 40–60, OR = 17.297 for those aged > 60, *p* < 0.001). We can further explore the potential differential associations of sedentary behavior with CVD across populations. For older adults, as mentioned, with advancing age, vascular function deteriorates, and the body′s ability to repair and adapt to stress decreases. Sedentary behavior can lead to reduced blood flow, increased blood viscosity, and the formation of blood clots more easily, which in turn increases the risk of heart attacks, strokes, and other cardiovascular events. For middle‐aged individuals, they often face high work and life pressure, and long‐term sedentary behavior may be associated with unhealthy lifestyle habits such as poor diet and lack of sleep, further exacerbating the risk of CVDs. For young adults, although their physical functions are generally better, excessive sedentary behavior, such as long‐term sitting in front of computers or mobile phones, can also lead to problems such as poor posture, reduced muscle strength, and metabolic disorders, which may have long‐term impacts on cardiovascular health. This finding suggests that older adults should be a primary target group for interventions aimed at reducing sedentary behavior. As individuals age, vascular function deteriorates, exacerbating the harmful effects of sedentary behavior. Previous research has similarly shown that increased sedentary time is associated with a higher risk of stroke, MI, and ACM, particularly among older adults [24]. For instance, it was found that every additional hour of sedentary time increases the risk of CVD or ACM by 33% (HR 1.33, 95% CI 1.14–1.56). Although light‐ and moderate‐intensity physical activity (LPA and MPA) can help reduce these risks, the adverse effects of sedentary behavior remain significant. Therefore, with advancing age, the detrimental effects of sedentary behavior become more evident, emphasizing the need for targeted interventions to promote physical activity among older adults.

Moreover, the study found a negative correlation between educational attainment and the impact of sedentary behavior. Individuals with higher educational levels demonstrated relatively lower risks of CVD, possibly due to their greater likelihood of receiving health education and engaging in more physical activities. Similar studies have shown that individuals with lower educational levels are more likely to overlook the health risks of sedentary behavior, thereby increasing their susceptibility to CVD. Improving education levels or raising public health awareness through targeted health promotion may be effective strategies to mitigate the adverse effects of sedentary behavior [25].

This study further analyzed the interaction between sedentary behavior and other cardiovascular risk factors. The results indicated that the impact of sedentary behavior on CVD risk was particularly pronounced among smokers and individuals with hyperlipidemia. This finding could be attributed to higher levels of metabolic abnormalities and inflammation in these high‐risk groups. Previous research has highlighted that sedentary behavior, when combined with traditional CVD risk factors such as smoking, hyperlipidemia, and diabetes, exacerbates the burden of CVDs [26]. Therefore, clinical interventions should focus on these high‐risk populations, employing combined strategies such as smoking cessation, dietary improvements, and reducing sedentary time to lower their CVD risks.

Given the results of this study, the public health significance of reducing sedentary behavior cannot be overstated. Research indicates that increasing physical activity not only effectively mitigates traditional cardiovascular risk factors but also alleviates the health risks associated with sedentary behavior through mechanisms such as improving autonomic balance, counteracting atherosclerosis, and promoting anti‐inflammatory effects [27, 28].

Despite uncovering important associations between sedentary behavior and CVD, this study has several limitations. First, its cross‐sectional design precludes the establishment of causal relationships. Secondly, in this study, sedentary time and CVD status were mainly based on self‐reported data. Self‐reported sedentary time may be subject to recall bias and social desirability bias. Additionally, defining CVD based on self‐reports and medication records may lead to misclassification (e.g., undiagnosed cases being categorized as healthy), which could make our effect estimates conservative or biased. Due to the limited total number of CVD cases, the results of subgroup analyses, especially those of certain subgroups, should be interpreted with caution. Their main value lies in indicating directions that need further validation in future large‐scale or prospectively designed studies. Future studies that combine device‐measured objective activity data with clinical diagnosis records will help validate these findings.

## 5. Conclusion

This study demonstrates that sedentary behavior significantly increases the risk of CVD, with the risk being especially high among older adults and individuals with lower educational levels. Moreover, sedentary behavior interacts with traditional cardiovascular risk factors such as smoking and hyperlipidemia, further exacerbating the burden of CVD. Therefore, reducing sedentary time and increasing physical activity are particularly crucial for high‐risk populations.

**Nomenclature**
CVD: cardiovascular disease
NHANES: National Health and Nutrition Examination Survey
SES: socioeconomic status
ACM: all‐cause mortality
NCHS: National Center for Health Statistics
CDC: Centers for Disease Control and Prevention
PIR: poverty‐to‐income ratio
BMI: body mass index
MCQ: multiple‐choice question
SBP: systolic blood pressure
DBP: diastolic blood pressure
NCEP: National Cholesterol Education Program
ATP III: Adult Treatment Panel III
HDL‐C: high‐density lipoprotein cholesterol
LDL‐C: low‐density lipoprotein cholesterol
RCS: restricted cubic spline
AIP: atherogenic index of plasma

## Author Contributions

Heming Huang, Zhe Zhao, Zemou Yu and Fan Feng contributed to the paper equally.

## Funding

The study was supported by the National Natural Science Foundation of China (No. 82400543).

## Ethics Statement

The authors have nothing to report.

## Conflicts of Interest

The authors declare no conflicts of interest.

## Data Availability

The data that support the findings of this study are available in National Health and Nutrition Examination Survey (NHANES) at https://wwwn.cdc.gov/nchs/nhanes/. These data were derived from the following resources available in the public domain: ‐ NHANES public datasets (various years), https://wwwn.cdc.gov/nchs/nhanes/Default.aspx
